# Scoping Review of Disease Surveillance Practices and Veterinary Care Use in Small-Scale Swine Farms in the United States

**DOI:** 10.3390/ani15111620

**Published:** 2025-05-30

**Authors:** Rachel A. Schambow, Michelle L. Schultze, Andres M. Perez

**Affiliations:** 1 Center for Animal Health and Food Safety, College of Veterinary Medicine, University of Minnesota, Saint Paul, MN 55108, USA; schu3298@umn.edu (M.L.S.); aperez@umn.edu (A.M.P.); 2 Department of Veterinary Population Medicine, College of Veterinary Medicine, University of Minnesota, Saint Paul, MN 55108, USA

**Keywords:** pig, swine, surveillance, veterinary use, African swine fever

## Abstract

The recent spread of foreign animal diseases (FADs) such as foot-and-mouth disease and African swine fever emphasizes the need to conduct comprehensive surveillance to detect a potential disease introduction as soon as possible. The United States is currently free of many important FADs of swine, and many preparedness initiatives have raised awareness amongst the commercial, intensive swine industry. However, the awareness and engagement of small-scale swine farmers regarding disease surveillance and passive reporting is not well known. This scoping review was conducted to identify and characterize sources of evidence on the practices and attitudes of small-scale swine farmers and owners in the United States regarding pig health and disease management, surveillance, and veterinary care use, and secondarily to characterize information seeking and communication behaviors. Sources of evidence were found through keyword searches of online databases, citation matching, and Sustainable Agriculture Research and Education project reports. Eligibility criteria included being conducted on or with US small-scale (defined in this review as less than 1000 pigs) or non-intensive swine farms and containing information pertinent to the objectives of the review. Seventeen sources were included in the final review. Regular disease monitoring and surveillance practices were not commonly reported, and multiple sources reported little to no incidence of disease occurrence in small-scale swine farms. Reported veterinary use and access was variable, and multiple sources reported that the choice to use veterinary care was affected by its perceived cost, value, and accessibility. Future research and outreach should aim to discern key factors affecting farmer’s decisions to use a veterinarian, improve their awareness and prioritization of swine diseases, and develop small-scale appropriate disease surveillance protocols. Ultimately, this will help small-scale swine farmers to protect the health of their pigs and improve FAD surveillance in the US.

## Simple Summary

In recent years, important diseases of pigs such as African swine fever and foot-and-mouth disease have been spreading into many new regions, posing significant threats to animal health, food security, and economic stability of affected regions. The United States is free of these diseases, but to help protect our pig industry from these threats, it is vital that we conduct comprehensive surveillance to detect a potential introduction as early as possible. Currently, disease surveillance practices and veterinary care use amongst small-scale swine farmers are not well known. Here, we conducted a scoping review to assess available sources of evidence. We found that few sources reported on disease surveillance practices, and that veterinary care use was limited amongst small-scale swine farms. Future research is needed to understand these practices amongst the variety of small-scale swine owners and to develop sustainable and targeted outreach strategies. Ultimately, this will help to improve the preparedness of the United States pig industry for high-impact swine diseases.

## 1. Introduction

The spread and impact of foreign animal diseases (FADs) such as African swine fever (ASF) and Foot-and-mouth disease (FMD) in recent years highlights the importance of preparedness to mitigate potential severe consequences of an FAD incursion [1,2,3]. Currently, the United States (US) is free of many important FADs that affect swine, including ASF, FMD, and classical swine fever (CSF) [1,2,4]. In a hypothetical 10-year scenario, it was estimated that an ASF introduction could cause losses of $50 billion, largely due to lost export markets [5]. Additionally, it was estimated that over 140,000 jobs could be lost, and that value-added could decrease by $11.1 billion. For a hypothetical FMD incursion, losses of around $100–200 billion have been estimated across all livestock industries, of which trade losses, indemnity, and direct production losses due to depopulation were the most substantial [6,7,8]. To help increase preparedness amongst the US swine industry, many national initiatives and programs have been developed, including Secure Pork Supply [9], the United States Swine Health Improvement Plan [10], a comprehensive ASF and CSF surveillance program operated by the United States Department of Agriculture (USDA) [11], National Pork Board’s AgView platform [12], and many more. However, the reach of these programs to small-scale and non-intensive swine farms is not well known.

Beginning in the early 1990s, the US swine industry experienced a significant structural shift. Farms became larger and more specialized with increased output, and the industry placed greater emphasis on pork exports [13]. From 1997 to 2017, the number of individual operations fell from about 120,000 to under 70,000 farms. Comparatively, the average number of pigs per farm grew from about 500 to nearly 1100 pigs. Currently, the majority of pigs in the US are raised in an intensive commercial setting, often characterized as large, integrated production systems with specialized production sites, indoor confinement housing, and increased efficiency of pig production [14,15,16]. Of the over 72 million pigs reported in the 2022 Census of Agriculture, 98% were raised in approximately 9800 sites with 1000 or more pigs [14]. However, there are vastly more individual small farms—over 50,000 swine farms with 999 pigs or less and over 43,000 farms with 24 pigs or less [14]. Additionally, in 2022, approximately 61% (over 34,000) of all farms with $1000 or more in agricultural sales that sold hogs were not specialized in hog production [17]. Small-scale swine farms do not have a specific or universal definition in the US. Generally, these farms have been referred to as small-scale, niche, alternative, extensive, backyard, non-commercial, regenerative, sustainable, heritage, and other terms depending on their attributes [18,19]. They may use a wide variety of management practices, from confinement housing to outdoor and pasture-access, organic, antibiotic-free, and more. Consequently, it is difficult to generalize across such a diverse group of farmers.

A strong surveillance system is critical for early detection and mitigation of FADs [20]. In particular, effective passive surveillance relies on having aware and active farmers across all types and sizes of livestock operations that can recognize important signs of disease and are willing and able to involve a veterinarian or make a disease report [20]. The potential gaps of awareness and implementation of these practices by small-scale swine farms and owners, along with appropriate channels for outreach, are not well known. To help direct future research and outreach efforts, an understanding of these aspects is needed. The objectives of this scoping review were to characterize the practices and attitudes of small-scale swine farmers and owners in the US regarding pig health and disease management, surveillance, and veterinary care use, and secondarily to characterize information seeking and communication behaviors. This work will help to direct future research and aid in the development of education and outreach to these groups regarding FAD surveillance and preparedness and ultimately help improve the strength of the US’s surveillance for swine FADs.

## 2. Materials and Methods

A protocol for this review was developed and is registered at the Open Science Framework (https://osf.io/mtuwf, accessed on 27 May 2025). This manuscript was prepared following the PRISMA Extension for Scoping Reviews guidelines [21].

### 2.1. Eligibility Criteria

Inclusion criteria for sources of evidence were those published in English and for which the full article/final project report was available and pertaining to US swine farmers or owners of any form of non-intensive, small-scale, outdoor-access, or non-commercial swine production. Small-scale was considered in this scoping review as the USDA, (2022) definition of 999 or fewer pigs [22]. Sources needed to include information on infectious and non-infectious disease and health management practices, infectious disease testing and surveillance practices, attitudes or awareness toward infectious and non-infectious diseases, veterinary care use and access, and/or knowledge-seeking and communication behaviors for swine-related information. All years of publication were included. Papers that focused solely on economic performance, housing, meat quality, pig performance, pig reproduction, biosecurity practices, pasture health, environmental health, or other aspects without any assessment or information relevant to the objectives of this review were excluded. For the same reasons, sources that focused on prevalence of swine diseases but did not contain any information on the targeted management practices were excluded. Sources conducted only in research settings and not with small-scale or non-commercial swine farms or owners were excluded as they would not represent real-world practices amongst these groups. Sources conducted on mixed-species farms or owners were excluded where no swine-specific information was reported. 

### 2.2. Information Sources and Search Strategy

All searches were conducted from February through March 2025. Because it was expected that relevant sources may exist outside of peer-reviewed literature, a multi-faceted search strategy was used. First, databases relevant to veterinary sciences and agriculture were searched using a structured search query. These included PubMed, Agricola, CAB Abstracts, Web of Science, Scopus, and agriRxiv pre-prints. The choice to use many databases was made because we expected that this type of information may be captured by multiple disciplines, including veterinary medicine, social sciences, agricultural sciences, and economics. After some initial testing of keywords for retrieved article relevance, the following search query was used with these databases without any additional filters:

((small or niche or pasture-raised or backyard or smallholder or extensive or alternative or organic or free-range or natural or outdoor or pasture) AND (“pig farm*” or “pig production” or “swine production” or “pork production” or “swine farm*”)) AND (“United States”)

In addition to structured databases, gray literature was also searched. CAB abstracts already index many gray literature sources such as conferences, proceedings, and theses. However, it was expected that some research or non-academic work would also be available through other agencies or groups relevant to small-scale farming. Potential sources including the USDA National Institute of Food and Agriculture and the USDA-APHIS National Animal Disease Preparedness and Response Program were considered but ultimately not used because project summaries or reports could not be obtained online for funded work. The Sustainable Agriculture Research and Education (SARE) program operates nationally in the US and has previously funded much work relevant to small-scale farmers [23]. A list of project reports from SARE were retrieved from their online database using the settings “All Project Types”, “All Years”, and Commodities set to “Hogs”. Finally, to supplement the previously described searches, both an ascendancy approach (looking at sources cited in the included published academic papers) and a descendancy approach (looking through sources that cited our included sources) were used.

### 2.3. Selection of Sources of Evidence

Source selection was conducted through multiple stages of screening. Because of the high number of retrieved sources, a first round of screening was conducted by reading titles according to the inclusion and exclusion criteria. Then, abstracts or project summaries from the retained sources were retrieved and reviewed. Finally, full papers or project reports were retrieved from the retained sources from the second stage of screening and read in full. Papers meeting the inclusion and exclusion criteria were retained for data extraction. This process was conducted for all sources (databases and gray literature). All screening was conducted by human reviewers (i.e., no artificial intelligence assistance). One reviewer reviewed all sources retrieved from databases, while a second reviewer reviewed all sources retrieved from citation matching and SARE. Throughout the screening process, both reviewers discussed any sources where it was unclear whether to retain them and came to a consensus decision. The screening process was conducted and tracked by downloading XLSX/CSV files from each database and SARE and using Microsoft Excel version 365 version 2501 and Zotero 7.0.11.

### 2.4. Data Charting and Synthesis

Data charting was conducted using a shared Google Sheet that was accessible to all team members. Each source of evidence was read in full, including all supplementary material. For each of the included sources of evidence, the following data items were charted: Year of publication, type of source (peer-reviewed article, government report, or post-grant project report), study design and methods used, whether it was swine-specific study or mixed species, a description of the swine farm/owner population, geographic location of farms/owners, the sample size or number of participants, veterinary care access and use, swine health management practices, disease monitoring and surveillance, disease prioritization and awareness, and knowledge-seeking and communication behaviors. Both quantitative and qualitative information were recorded where available. Additionally, a column was used for any comments regarding potential bias or evaluation of the source, though a systematic, critical appraisal was not performed. One team member independently charted information for all sources with discussion of findings with the other team members.

For two sources (Baye et al. (2024) and Lee et al. (2022)), compiled questionnaire response data were available as a supplemental XLSX or CSV file [24,25]. These datasets were especially relevant because Baye et al. (2024) reported on both large and small swine farmers, while Lee et al. (2022) reported on backyard and small commercial owners of multiple food animal species. For both datasets, it was possible to select only those observations from small-scale swine farmers. These datasets were accessed, downloaded, and descriptively analyzed using PivotTables in Microsoft Excel to retrieve relevant information in a manner similar to that reported in their respective peer-reviewed articles but stratified to small-scale swine farms. No data cleaning or modifications were made from the downloaded datasets.

Data were synthesized by examining and grouping all collected information within each charting category. General characteristics of each source were summarized. Data items relevant to the objectives of the review (veterinary care access and use, swine health management practices, disease monitoring and surveillance, disease prioritization and awareness, and knowledge-seeking and communication behaviors) were grouped by topic and reviewed. Where quantitative estimates were reported on similar topics (e.g., vaccination use and having a veterinarian), these values were summarized together as a range or represented in comparative tables. Otherwise, quantitative estimates were simply reported in their relevant sections. Where qualitative information was available, this information was summarized, grouped by similar topics where possible (e.g., barriers to veterinary access and qualitative observations from co-authors or project teams), and reported in their relevant section.

## 3. Results

### 3.1. Selection of Sources of Evidence

**Figure 1:**
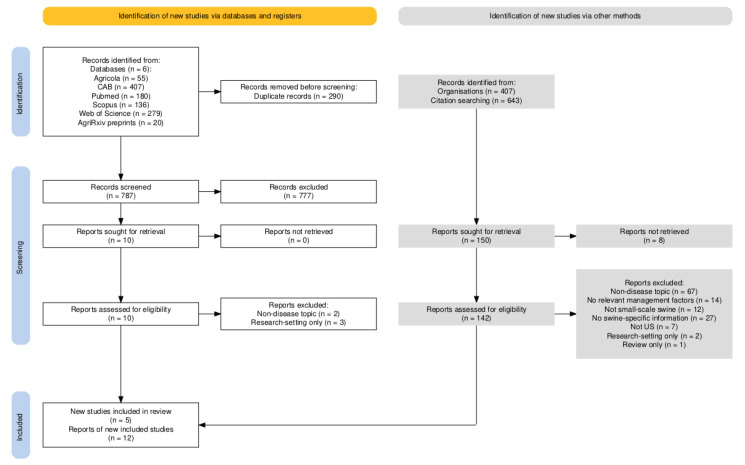
PRISMA flow diagram of the numbers of sources of evidence screened, assessed for eligibility, and included in the review [26].

A PRISMA flow diagram outlining the process of source selection is available in Figure 1 [26]. From databases, 1077 sources were found. There were 787 unique sources (290 duplicates removed). After screening titles, 81 were selected for review of abstracts. Reasons for exclusion during this stage were not specifically tracked, but included not being related to swine production, not being related to the United States, being focused on biomedical research (diagnostic test production, vaccine production), being focused on housing or environmental management, and not being available in English. From the 81 selected sources whose abstracts were reviewed, 10 sources were retained; reasons for exclusion were similar to those for the title screening stage. After reviewing the selected full papers, five of these were included in the final scoping review. Full papers were excluded because they focused on housing (n = 1), research-setting only (n = 3), or focused on marketing and economics (n = 1).

Because work was occurring simultaneously by two reviewers, all citations and papers who cited them were reviewed from the ten full papers that were reviewed, not just the five included in the final scoping review. In total, 643 citations were reviewed after removal of duplicates (n = 162 duplicates removed). After screening the titles, abstracts from 188 sources were reviewed. Sixty sources were selected for review in full. Seven were included in the final scoping review. Reasons for exclusion included the following: n = 13 non-disease topic, n = 7 no relevant management factors reported, n = 12 not small-scale swine, n = 12 no swine-specific information, n = 7 not US, n = 1 research-setting only, and n = 1 review only.

From SARE, 407 project sources were found. Project summaries from 90 sources were selected for full review after title screening. Eight did not have a final project report or were incomplete projects. Five project reports were included in the final scoping review. Reasons for exclusion included the following: n = 54 non-disease topic, n = 7 no relevant management factors reported, n = 15 no swine-specific information, and n = 1 research-setting only.

### 3.2. Characteristics of Selected Sources of Evidence

Seventeen sources of evidence were included in the final scoping review (Table 1). These included nine peer-reviewed articles, five post-grant reports from SARE-funded projects, and three USDA reports. Sources were from 2007 to 2024. Four sources (all peer-reviewed articles) assessed owners with multiple species, such as poultry, cattle, small ruminants, and camelids, in addition to swine. All sources focused on or included small-scale or non-commercial swine owners except for Pires et al. (2020), who studied veterinarian’s engagement with urban and peri-urban poultry and livestock owners [27]. Studied populations were described as small-scale, backyard, alternative, outdoor access, organic, and niche swine farms. Geographically, sources focused mainly on the northeast (PA, NY, NH, ME, MA, CT), the west (CA, OR, CO, WA), the midwest (IA, MN, IL, NE, and KS), or were conducted nationally. No sources were found that focused on southern or southwestern states. For sources that assessed swine farms (omitting the sources on miniature swine owners [28], youth exhibition swine [29], and veterinarians [27]), the maximum herd size of farms assessed was 999 pigs. Eleven sources had farms with maximum sizes of less than 500 pigs, and nine with 100 or fewer pigs. Reports from USDA used different categorizations over time [22,30,31]. USDA, (2009) categorized farms as small (1–24 pigs), medium (25–49 pigs), and large (50–99 pigs). USDA, (2014) did not use specific labels, but separately reported on farms with 1 to 49 pigs and farms with 50 to 99 pigs. USDA, (2022) categorized them as very small (1–24 pigs), small (25–49 pigs), medium (50–99 pigs), large (100–499), and very large (500–999 pigs).

### 3.3. Health Management Practices

Vaccination, antibiotic use, and deworming were assessed by many of the included sources. Vaccination practices varied and depended on the type of swine farms studied [22,29,33,34,36,40]. Midwest niche swine farms studied by Yaeger et al. (2009) widely used vaccination for breeding animals, with 91% of farms vaccinating for porcine parvovirus, Leptospira interrogans serovars, and Erysipelothrix rhusiopathiae [40]. Vaccination rates amongst suckling pigs varied from 18 to 55% depending on the pathogen. A total of 27% of farms did not vaccinate their nursery pigs, and 64% did not vaccinate finishing pigs. From Wayne et al. (2012), 30% of Minnesota 4-H youth exhibitors reported that their pigs had been either vaccinated against or treated for PRRS [29]. Similarly, 29% of Midwest (mainly Minnesotan) small farms reported using vaccines [36], though vaccine types were not specified. All northeastern farms studied by Hurwitz and Delaney, (2023), reported using vaccinations, ranging from 21% of farms vaccinating their market hogs to 83% of farms vaccinating their piglets or feeder swine [34]. Thirty-eight percent of farms studied by USDA, (2022) practiced vaccination [22]. By category, this ranged from 30% (very small) to 73% (very large). Regionally, vaccination was highest in the Central region (57%) and lowest in the Northeast region (26%). Similarly, Havas et al. (2022) reported that vaccination was limited on the New York farms that they sampled [33].

Antibiotic use was variable [22,25,31,34,38]. Of farms reported on by USDA, (2022), the use of antibiotics in feed or water for sows or gilts was overall low and ranged from 2.5 to 26% by size category. For weaned pigs, this ranged from 36.1–56.8% in 2012 to 1.6–40% in 2021 [22,31]. Generally, antibiotic use increased with larger farm size categories. Of western swine owners included in Lee et al. (2022), 17 (46%) purchased antibiotics and 19 (51%) had used them in the previous year [25]. Across different health concerns, they mainly treated only the affected animal, rather than group treatment. The health concerns that owners were more likely to treat included respiratory, digestive/scours, lameness, eye, or reproductive while mastitis, weak newborns, or not eating. Similarly, 57% of northeastern farms from Hurwitz and Delaney, (2023) had purchased “over the counter” antibiotics [34]. Three small-scale farms (20%) studied by Osadebe and Heimer, (2010) used antibiotics, two in water for 8–10-week-old piglets and the remaining farm only during winter months when pigs were housed indoors [38].

Compared to vaccination and antibiotic use, deworming was a more common practice in the studied populations [22,34,35,39,40]. On niche swine farms in the Midwest, 91% reported dewormed sows, 82% dewormed nursery pigs, and 64% dewormed finishing pigs using ivermectin, dichlorvos, and/or fenbendazole [40]. Exner et al. (2007) did not report on deworming practices, but did comment that some farms had high parasite levels [32]. The majority of northeastern herds that participated with Hurwitz and Delaney, (2023) also were affected by internal parasites, and 21–71% reported deworming pigs, depending on the production category [34]. Of farms studied by USDA, (2022), 71% practiced deworming overall, ranging from 65% (very small farms) to 93% (small farms) by category [22]. A total of 5 out of 9 studied organic farms reported using non-synthetic anthelmintics such as apple cider vinegar, diatomaceous earth, garlic, and a commercially available herbal deworming powder [35]. Though not swine-specific, 97% and 93% of backyard farm respondents were at least somewhat comfortable with deworming [39]. In contrast to these reports, Havas et al. (2022) commented that parasite management was limited in New York swine farms, though a specific percentage was not reported [33].

### 3.4. Disease Monitoring, Surveillance, and Diagnosis

Where it was described, disease monitoring, surveillance, and diagnosis varied considerably. A total of 50% of northeastern farms studied by Hurwitz and Delaney, (2023) reported they had not experienced a disease outbreak in the past three years, and 14% reported they were not sure [34]. Similarly, in USDA, (2009), 77.8% of farms report no deaths of any pigs during the previous twelve months, and only 7.6% had any pigs show signs of unusual diseases [30]. From Hurwitz and Delaney, (2023), only 7% reported they experienced a disease outbreak and had not diagnosed the cause [34]. A total of 57% reported that they performed or had someone perform necropsies for unexplained pig mortalities. Conversely, Nicholoson et al. (2022) reported that 82% of 105 Pennsylvanian respondents (which included poultry owners) never submitted a dead animal to the state veterinary laboratory for disease confirmation, and Marshall et al, (2007) reported that 62% of miniature pig owners would be unlikely to request a necropsy if their pig died [28,37].

Specific clinical signs or syndromes, as identified by farms, were reported by five sources [22,30,31,32,36]. In USDA, (2009), difficulty breathing was the most commonly reported sign across farm sizes but was still generally low (3.3%) [30]. In USDA, (2014), farms reported signs associated with specific diseases, of which roundworms (8.2%), diarrhea (4.3%), and Mycoplasma pneumonia (3.1%) were most common [31]. Farms studied by USDA, (2022) most commonly reported “found dead or reason unknown” (20%) compared to other disease problems across production stages, specifically affecting 10.6% of farms’ sows and gilts [22]. Respiratory disease was most common in nursing, nursery-aged, and grower/finisher pigs (9–11%), while sows most commonly experienced reproductive problems (19%). Internal and external parasites affected 7.1 and 5% of all pig types on farms, respectively. In the same report, disease testing for surveillance (specifically not to diagnose sick animals) was low for very small farms (1.9% for pseudorabies virus and 1.8% for brucellosis), but ranged from 15 to 23% for small to very large farms. Twelve Midwest swine farms studied by Medrano et al. (2023) had a previous diagnosis of infectious diseases, including Erysipelas, exudative epidermitis, PRRS, influenza, circovirus, and roundworms, suggesting some previous disease testing was occurring in these farms [36]. Exner et al. (2007) generally described that the surveyed Midwest niche swine farmers tracked piglet death loss in detail, including recording the litter, age of dead piglets, and apparent cause of death [32]. The most common causes of post-weaning mortality, according to farmers, were Salmonella, ileitis, and scours. However, similar to USDA, (2022), they also reported that “unknown” was a frequently cited cause of death [22].

Some sources reported specifically on quarantine and disease testing procedures for bringing in new animals to a farm’s herd [22,33,34,36]. According to USDA, (2022), for farms that had introduced new sows or gilts, 79% always quarantined them [22]. However, only 15% of very large farms reported always quarantining new sows or gilts. Disease testing prior to introduction was overall low (28%), ranging from 15% in very large farms to 37% in medium farms. Regionally, this was highest in the Central, South, and East regions (49%, 30%, and 27%, respectively), and very low in the West and Northeast regions (4.3% and 2.4%, respectively). Almost all New York swine farms studied by Havas et al. (2022) purchased new pigs from farms without knowing the animal or farm’s brucellosis and pseudorabies disease status [33]. A total of 5 out of 18 (28%) farms that purchased breeding pigs and 8 (44%) that purchased non-breeding pigs reported some pre-purchase health requirements, including inspection of visual appearance, vaccination or deworming, use of state guidelines, and breed standards. Only one breeding farm reported purchasing pigs from a closed herd that provided health records. Regarding quarantine, 16 (89%) farms isolated new pigs for 7–30 days, 7 of which isolated new pigs for 30 days. Similarly, 12 of 14 (86%) northeastern swine farms reported some form of quarantine for new and sick pigs [34]. However, the investigators noted that upon field visits, few farms were able to implement best practices or true quarantine.

Some sources also reported on farmers’ attitudes and awareness regarding swine infectious diseases. Half or more of 14 northeastern farmers studied by Hurwitz and Delaney, (2023) felt informed about and able to address internal parasites (93%), external parasites (71%), porcine parvovirus (50%), and Mycoplasma hyopneumoniae (50%) [34]. Only one and two respondents felt informed about FMD and ASF, respectively. Similarly, in USDA, (2014), only 11.9% and 18.8% of respondents felt very or extremely familiar with CSF and FMD, respectively [31]. Across multiple sources, swine influenza was commonly reported as the disease that respondents were most or very familiar with, or recognized as very important [29,31,34,37]. Other farmers showed high interest in understanding the risks and management of gastrointestinal parasites [38]. Seventy-nine percent of farms from Hurwitz and Delaney, (2023) were somewhat confident that their farm was well protected from contagious diseases [34]. One and zero farmers considered ASF and FMD as a risk to their farms, respectively. When presented with an example scenario of a potential ASF outbreak, 62% of small farms agreed or strongly agreed that ASF would be a risk to their operation, while 13% disagreed or strongly disagreed [24].

Additionally, some barriers to regular testing were identified. All small-scale northeast farms from Hurwitz and Delaney, (2023) reported that they would want to continue disease surveillance testing if full funding were provided [34]. However, only 20% would be willing to continue without external funding and 80% reported “Maybe”. Barriers identified by these participants included cost (all participants), containing or handling pigs for sampling, and not knowing who could conduct sampling.

### 3.5. Veterinary Care Use and Access

Nine sources reported on veterinary care use and access in small-scale swine farms or non-commercial owners (Table 2). Where having a veterinarian was not a requirement for study enrollment, the percentage of respondents or sampled farms that had a veterinarian or had consulted with a veterinarian ranged from 25 to 86% [22,25,28,30,31,33,34,36]. Common uses for veterinarians from Lee et al. (2022) included phone or email consultation, regular or routine visits, for antibiotics, and emergency calls and sick animal care [25]. In Nicholson et al. (2020), backyard swine owners reported they would seek immediate help most commonly for future cases of diarrhea (about 95% of owners), difficulty breathing (about 95%), and fever (about 90%) [37]. This was less for other signs (60–73% of owners), including difficulty walking, pigs being extremely tired, hemorrhages/red skin, high death loss, not eating, and sores/ulcers. Other reported reasons for using a veterinarian included obtaining certificates of veterinary inspection and a veterinary feed directive prescription [25]. Perceived lack of utility, need, or skepticism for the value of regular preventative veterinary care (as opposed to emergency or sick animal care only) was noted in multiple sources [32,33,34,39]. Pires et al. (2019) did not report specifically on swine owners, though about 25% of the respondents had swine on their farm [39]. Of these 351 western poultry and livestock owners that reported an animal health concern in the previous twelve months, under half called a veterinarian. Seventy-five percent knew of a veterinarian who treated livestock. Reported reasons for not contacting a veterinarian included managing the concern on their own, cost, and the availability of veterinarians with livestock experience. Similarly, 34.3% of farms sampled in USDA, (2009) said they sought a veterinary or diagnostic lab for assistance with unusual clinical signs, while 40.4% self-treated pigs [30]. Trouble accessing a veterinarian to treat swine was directly commented on by a small number of participants in three sources [25,33,36].

Regarding the intention to call a veterinarian for a herd exam in the presence of clinical signs of ASF in a scenario where ASF is likely to be detected, 67% of small swine farms agreed and 22% strongly agreed they would do so [24]. Only 8% were unsure, and 3% disagreed or strongly disagreed. Regarding readiness to report infection, 84% agreed or strongly agreed, 14% were unsure, and only 2% disagreed and strongly disagreed.

Pires et al. (2020) assessed veterinarians’ engagement with backyard and small-scale poultry and livestock owners in urban and peri-urban areas in western states [27]. Of 808 sampled veterinarians, 10.9% reports examined >2 swine in the previous six months. Approximately 83% said they were not likely to examine swine, and only about 9% said they were likely or very likely to examine swine. Over half reported lack of interest as the main reason for not examining swine. Other reasons included financial constraints for owners, lack of equipment or facilities, lack of demand, or being a species-limited practice. Over half agreed or strongly agreed that there were sufficient veterinarians in their practice area to treat production animal species.

### 3.6. Knowledge Seeking and Communication

Main or very important sources of information on swine production and animal health included the Internet (ranging from 23 to 82% of respondents) [22,30,34,37,39], veterinarians (45–62%) [22,30,32,34,37,39], friends, neighbors, other pig owners, other community members (35–55%) [22,30,34,37,39], and feed or animal health product suppliers (48–49%) [22,30]. The lowest value reported for Internet use (23%) was reported by USDA, (2009), while the remaining values from 2019 to 2023 were 50% of respondents or higher. Other less commonly specified sources were government departments, local animal clubs or organizations, university agriculture extension, pork industry publications, programs or meetings, and producer web discussions via social media and other platforms [22,30,34,37,39]. Pires at al. (2019) reported that participants searched for animal health information on a monthly basis and searched for procedure and treatment-specific information on a yearly basis [39]. Of respondents from Baye et al. (2024), 87% of small swine farms reported that they had attended at least one disease eradication program [24]. These included Secure Pork Supply, brucellosis, porcine epidemic diarrhea virus, pseudorabies, and other programs. Fifty-four percent of Minnesota 4-H youth reported that they primarily participated in the swine project to learn about pigs and livestock husbandry [29].

Sixty percent of farmers that participated in the study by Hurwitz and Delaney, (2023) reported they belonged to an industry association, including Farm Bureau, A Greener World, Maine Pork Producers, American Berkshire or National Berkshire Association, University of New Hampshire Extension Advisory Board, Granite State Graziers, and Maine Grass Farmers Network [34]. These farmers also reported that they were interested in seeking information from discussion groups facilitated by swine specialist or industry associations, offerings by state organizations, or regular consultations with private practice veterinarians.

Knowledge-seeking by veterinarians regarding small-scale swine and other production species was also assessed by Pires et al. (2020) [27]. Amongst the sampled veterinarians, 65.5% reported they had little knowledge for answering clients’ questions pertaining to swine husbandry, and most said they had little or basic knowledge on livestock restraint, exam, and disease diagnosis. Regarding accessing further training or education, only about 28% actively looked for continuing education options for backyard poultry or livestock and about 36% would be interested in participating in a continuing education program focused on production animal species. Interest in swine-specific training was also reported to be low. Factors that were significantly associated with an interest in livestock-focused continuing education programs included receiving more livestock questions from clients, examining livestock, being in rural areas compared to urban, having existing knowledge in livestock exam procedures, and practice type other than companion animal only.

## 4. Discussion

This scoping review identified sources of evidence related to disease management and surveillance practices, veterinary care use and access, and knowledge-seeking and communication behaviors of small-scale swine farmers and owners. The included sources that cover a wide type and size of populations, though over half described swine farms with 100 or fewer pigs. This is consistent with the majority of small-scale swine farms in the US having relatively few pigs [14]. Only three of the peer-reviewed sources conducted work directly with small-scale swine farms [29,33,40]. This may be due to the lack of resources or interest for academic works on small-scale swine compared to increased focus on biosecurity practices or small-scale poultry. Additionally, no sources were found that focused on small-scale swine farms in southern US. The reason for this is unclear, but it may be influenced by the relatively low level of intensive swine production in this region [14].

Information from the identified sources on health management practices was very limited and highly general. Pathogen-specific management practices were not reported. Instead, general information was found on vaccination practices, antibiotic use, and deworming practices. Deworming was common, which is expected considering that pigs in bedded hoops or with outdoor access may be subject to higher parasite pressures, though specific parasites were not mentioned [41]. Antibiotic use was very variable, which is not surprising considering that many small-scale farms may raise and market pigs as antibiotic-free [18,19]. The sources did not describe diagnostic procedures used to support the choice of antibiotics, nor were pathogens specified for which antibiotics were used. Considering that vaccination is a regular preventative measure across veterinary species and is allowed in organic production with some limitations [42], it is not surprising that this was also commonly reported, though to a lesser extent than deworming. Parasite management and vaccination programs could be opportunities to connect small-scale farms with a regular veterinarian for ongoing and valuable preventative care.

Specific disease monitoring and surveillance practices were mostly absent from the included sources and mainly reported in the context of adding new pigs to the herd. Quarantine procedures were more commonly reported, but consistent with an observation by Hurtwitz and Delaney, (2023), the definition and facilities for quarantine used by those farms may not be adequate for true quarantine and prevention of disease spread [34]. Notably, some of the sources indicated that deaths with unknown causes and without further necropsy or diagnostic workup were not uncommon. This is a challenge for FAD surveillance, especially considering that in relatively small herds, normal indicators of a serious problem such as “high mortality” may be difficult to apply. Cost was reported as a barrier to regular disease testing, further suggesting some small-scale farmers may not see a favorable cost–benefit relationship, even if they recognize the importance of disease testing and have access to a veterinarian. Considering that many swine farmers recognized and were concerned about swine influenza, this priority could be aligned with education and awareness on swine disease surveillance reporting to help strengthen passive surveillance systems.

Veterinary care use and access was reported by the majority of sources. This is consistent with recent, growing concerns about food animal veterinarian shortages and accessibility [43,44]. The percentage of farms with a veterinarian varied substantially across sources (Table 2). While challenges with veterinary access were documented in multiple sources, it did not appear to be the only or, in some scenarios, the most important factor affecting swine farmers’ choice to use a veterinarian. The perceived value and economic return of veterinary care, particularly regular preventative care, may be as influential as veterinary accessibility. This might be especially true for small farmers, who can have reduced economic efficiency compared to intensive production [19]. The cost of regular veterinary visits might not result in an economically favorable increase in productivity or reduction in mortality for these farms. This is concerning for many reasons, as veterinarians are essential for providing reliable information to small-scale farmers on swine health and recognizing important signs of potential FADs. This may be further compounded by the fact that in many sources, farms reported little to no incidence of disease in their pigs. Parameters used in intensive commercial production to alert swine farmers to the need to call a veterinarian, such as weekly mortality or morbidity, may not be easily transferred to a small-scale production setting, indicating the need for the development of surveillance protocols tailored for small populations. For small farms, basic disease surveillance can be conducted economically through daily observation of pigs and recording clinical signs. This should be conducted for all pigs on small farms or at group or pen-level on larger farms. Currently, there are numerous resources available online to help swine owners with recognizing and reporting important clinical signs of swine FADs [45,46,47]. A record of signs can help farmers to track and understand their pigs’ health status over time and may be kept via paper or electronically, for which free or economical software like word processors or spreadsheets can be used. The success of this type of approach, however, depends on the ability of farmers to recognize clinical signs and their willingness or ability to make a disease report or contact a veterinarian, for which further work is likely needed.

This scoping review revealed many gaps about our current understanding of disease management, surveillance practices, and veterinary care usage by small-scale swine farms and owners. It is essential to understand the motivating and demotivating factors affecting farmers’ use of professional veterinary care to develop solutions that address complex factors such as cost–benefit perception and veterinary accessibility. Economic evaluations are needed to understand financial reasons influencing the use of veterinary care and surveillance protocols, and to identify ways to make regular care affordable and accessible. Farmers’ knowledge, awareness, and perceived importance of swine infectious diseases, including FADs, is also needed and will help in tailoring outreach and education programs in ways that match farmers’ priorities and interests, ultimately improving engagement with programs. For example, if many farmers are concerned about managing parasites, programs on clinical sign detection and disease monitoring could be centered around these issues, with transferability of skills to general swine care and FAD recognition. Because it may be difficult to recruit small-scale farmers, studies of swine veterinarians, similar to those conducted by Pires et al. (2020), could help in understanding their engagement with small-scale swine farms and the health status of and practices implemented on farms they work with [27]. Some common channels of communication were identified here, such as through veterinarians, feed or animal health product representatives, or the Internet; but given the diversity of responses, it is critical that these are better understood so that information can be delivered to the intended recipients. Across these gaps, it is also important to recognize the diversity of small-scale swine farms and to avoid generalizing information from one subgroup to another. For example, small-scale swine farmers in the Northeast may have different practices and awareness compared to Midwestern farms that might ultimately affect disease control, risk communication, and compliance. Other differences may exist depending on factors such as being antibiotic-free, organic, heritage breeds, and other factors.

There were some important limitations of the review. Many sources had a low sample size, which likely reflects the common difficulty for researchers and education specialists in accessing or engaging with small-scale swine farmers. For most sources, the factors of interest for this review were not the source’s primary focus. Consequently, the style of reporting was not consistent across sources. It is also possible that some informal sources, such as industry or association reports, may not have been captured here as they are not likely to be indexed in databases. Additionally, five sources were project reports from SARE-funded projects, thus they were not subject to a regular, peer-review process. However, all project proposals were reviewed and funded through SARE’s regular and rigorous grant process, and they represented important information on small-scale swine that was not available in other formats.

## 5. Conclusions

This review provides foundational information on disease management, surveillance practices, and veterinary care use and access amongst small-scale swine farmers. Disease monitoring and surveillance do not appear to be common practices in small-scale swine farms, which suggests the need for specific training and awareness that aligns with farmers’ needs and priorities. Reported veterinary use and access was higher than expected in some sources but still demonstrated a critical need to encourage and help farmers to access and invest in regular, preventative veterinary care. Future research and outreach should focus on understanding the factors that affect a farmer’s decisions to use a veterinarian, their awareness and perception of swine diseases, and enhance their disease surveillance and monitoring skills. Ultimately, this will help small-scale swine farmers to enhance the health of their pigs and improve the strength of FAD surveillance in the US.

## Figures and Tables

**Table 1:** Sources of evidence included in the scoping review.

Reference	Source Type	State/Region	Species Focus	Population Focus	Size of Swine Farms	Study Focus
Baye et al. (2024) [24]	Peer-reviewed article	National	Swine	Large and small-scale swine producers (<500 pigs)	Small farms categorized as <500 pigs	Attitudes toward biosecurity and disease reporting
Exner et al. (2007) [32]	SARE Project	IA	Swine	Alternative swine farms	Not specified	Piglet mortality assessment and outreach
Havas et al. (2022) [33]	Peer-reviewed article	NY	Swine	Swine farms with outdoor access	≤42 nonbreeding pigs, ≤11 sows	Serological study of Brucella suis exposure, and assessment of biosecurity and management practices
Hurwitz and Delaney, (2023) [34]	SARE Project	ME and NH	Swine	Small-scale swine farms	Herd size 30–650 pigs	Biosecurity and infectious disease prevention
Lee et al. (2022) [25]	Peer-reviewed article	CA	Multiple Species	Small-scale and backyard farms	92% with 1–5 pigs, maximum 21–50 pigs	On-farm husbandry, disease prevention, biosecurity, and antimicrobial use
Major, (2024) [35]	SARE Project	PA	Swine	Organic swine farms	10–60 pigs raised annually	Prevalence of gastrointestinal parasites
Marshall et al. (2007) [28]	Peer-reviewed article	National	Swine	Miniature swine owners	70% with 1–2 pigs, maximum 39 pigs	Management practices and veterinary use
Medrano et al. (2023) [36]	SARE Project	MN, and IL and WI	Swine	Small-scale swine farms	Growing 4–490 pigs, breeding 1–48 sows	Disease prevalence and assessment of management practices
Nicholson et al. (2020) [37]	Peer-reviewed article	PA	Multiple Species	Backyard poultry and swine owners	Median 3 pigs	Zoonotic disease awareness, veterinary care use, and biosecurity practices
Osadebe and Heimer, (2010) [38]	SARE Project	PA, MA, CT	Swine	Small-scale swine farms	75% with ≤25 pigs, maximum 99 pigs	Prevalence of Clostridium difficle
Pires et al. (2019) [39]	Peer-reviewed article	CA, OR, CO, and WA	Multiple Species	Urban and peri-urban small-scale and backyard livestock and poultry owners	Maximum 100 pigs	Veterinary service needs, management and husbandry regarding disease prevention, and attitudes on animal health and food safety
Pires et al. (2020) [27]	Peer-reviewed article	CA, OR, CO, and WA	Multiple Species	Veterinarians	Not applicable	Veterinary engagement with poultry and livestock owners in urban and peri-urban areas
USDA, (2022) [22]	USDA Report	National	Swine	Small-scale swine farms	Majority (44%) ≤24 pigs, maximum 999 pigs	Inventory and management practices
USDA, (2014) [31]	USDA Report	National	Swine	Small-scale swine farms	<100 pigs	Inventory and management practices
USDA, (2009) [30]	USDA Report	National	Swine	Small-scale swine farms	<100 pigs	Inventory and management practices
Wayne et al. (2012) [29]	Peer-reviewed article	MN	Swine	4-H swine youth/exhibition swine	Not applicable	Serological study of porcine reproductive and respiratory syndrome virus in 4-H exhibition swine and assessment of youth swine knowledge and management practices
Yaeger et al. (2009) [40]	Peer-reviewed article	IA, MN, IL, NE, and KS	Swine	Niche swine farms	Average 70 sows, 30–200+ sows	Disease prevalence and management

**Table 2:** Veterinary care and access in sources that assess swine specifically. CVI = certificate of veterinary inspection. VCPR = veterinary client patient relationship.

Study	Frequency of Use	Types of Care Used	Barriers to Access
Havas et al. (2022) [33]	16 out of 20 (80%) had any type of veterinarian	Sick animal care only (n = 13), general health purposes (n = 3); preventive medicine programs such as vaccination and parasite management were limited	Absence of veterinarians that treat swine in the region (n = 1)
Lee et al. (2022) [25]	23 out of 28 (82%) used a veterinarian, and 15 out of 24 (62.5%) had a VCPR	Consulted over phone or email (n = 15), regular or routine visits (n = 13), emergency calls (n = 10), CVIs (n = 4), 4 for other, and 1 for feed VFDs and water prescriptions	Trouble having a veterinarian to come to farm (n = 2)
Yaeger et al. (2009) [40]	26 out of 26 (100%) had a veterinarian with swine expertise (requirement of enrollment)	Not reported, but local veterinarians had to agree to participate in the study. Farms also regularly used vaccination and deworming protocols	Not reported
USDA, (2022) [22]	61% of all farms had VCPR, 56% of very small, 70% of small, 85% of medium, 77% of large, and 98% of very large	Not reported, however, veterinarians were listed as a important source of information	Not reported
USDA, (2014) [31]	25.2% had 1 or more visits from a local veterinarian in the previous year	Not reported	Not reported
USDA, (2009) [30]	29% had 1 or more visits from a local veterinarian in the previous year	To investigate unusual clinical signs or mortality >10%, and as a very important source of information	Not reported
Hurwitz and Delaney, (2023) [34]	10 out of 14 had a veterinarian (71%)	Not specifically reported, but farms used vaccination, deworming, and antibiotics, and had necropsies performed, and used veterinarians as a source of information	Perceived lack of need or utility by owners more than lack of access, but also some evidence of lack of swine practitioners
Medrano et al. (2023) [36]	55% had consulted a veterinarian	Not reported	Difficulty in accessing veterinary care (n = 2)
Marshall et al. (2007) [28]	At least 91 of 106 (86%) respondents (exact number not specified)	Median of 1 veterinary visit per year per respondent	Not reported

## Data Availability

No new data were created or analyzed in this study. Data sharing is not applicable to this article.

## Abbreviations

The following abbreviations are used in this manuscript:

ASF	African swine fever
CSF	Classical swine fever
FAD	Foreign animal disease
FMD	Foot-and-mouth disease
SARE	Sustainable Agriculture Research and Education
US	United States
USDA	United States Department of Agriculture
USDA APHIS	United States Department of Agriculture Animal and Plant Health Inspection Agency
